# Periacetabular Osteotomy and Postoperative Pregnancy—Is There an Influence on the Mode of Birth?

**DOI:** 10.3390/jcm11164836

**Published:** 2022-08-18

**Authors:** Friederike Schömig, Christian Hipfl, Jannis Löchel, Carsten Perka, Sebastian Hardt, Vincent Justus Leopold

**Affiliations:** Center for Musculoskeletal Surgery, Charité—University Medicine Berlin, Charitéplatz 1, 10117 Berlin, Germany

**Keywords:** developmental hip dysplasia, periacetabular osteotomy, cesarean section, pregnancy complications

## Abstract

As a surgical treatment option in symptomatic developmental dysplasia of the hip, periacetabular osteotomy (PAO) is often performed in female patients of childbearing age. Yet, to date, little is known about the procedure’s influence on postoperative pregnancies and the mode of delivery. Our study’s aim therefore was to investigate patient and physician decision making in women after PAO. We invited all patients who had undergone PAO in our institution from January 2015 to June 2017 to participate in a paper-based survey. Of these, we included all female patients and performed a retrospective chart review as well as analysis of pre- and postoperative radiological imaging. A total of 87 patients were included, 20 of whom gave birth to 26 children after PAO. The mean overall follow-up was 5.3 ± 0.8 years. Four (20.0%) patients reported that their obstetrician was concerned due to their history of PAO. The mean time before the first child’s birth was 2.9 ± 1.3 years. Eleven (55.0%) patients underwent cesarean section for the first delivery after PAO, three of whom reported their history of PAO as the reason for this type of delivery. Patients with a history of PAO have a higher risk of delivering a child by cesarean section compared with the general population, in which the rate of cesarean section is reported to be 29.7%. As cesarean sections are associated with increased morbidity and mortality compared with vaginal deliveries, evidence-based recommendations for pregnancies after pelvic osteotomy are needed.

## 1. Introduction

Developmental dysplasia of the hip (DDH) is defined by an insufficient acetabular coverage of the femoral head and is a leading cause of secondary hip osteoarthritis [1,2]. Despite the existing strategies for early detection and thereby early treatment, DDH continues to be diagnosed at older ages. In the case of symptomatic DDH without signs of osteoarthritis, surgical management is an option in skeletally mature patients to preserve the native hip joint [3].

As one of the surgical techniques performed in the treatment of DDH, periacetabular osteotomy (PAO) allows for a three-dimensional reorientation of the hip socket to provide improved coverage of the femoral head. This is achieved by completely detaching the acetabulum from the pelvis through defined osteotomies along the Os ischium, Os pubis and Os ilium before fixing the acetabular fragment either with K-wires or screws [4,5,6,7]. In contrast to other pelvic osteotomies, the posterior column remains intact [4]. Previous studies have shown good to excellent outcomes both clinically and radiologically for this procedure [3,6,8,9,10].

As DDH occurs predominantly in women, and mostly younger patients become candidates for hip preservation surgery, PAO is mostly performed in a population of female patients of childbearing age [11]. Previous reports have shown differing results regarding changes in birth canal morphology after different types of pelvic osteotomies. While a study by Ishimatsu showed that 17% of patients had birth canal diameters under the cutoff value for increased risk of the need for a cesarean section after curved periacetabular osteotomy [12], other analyses found no significant changes in birth canal diameters in women after PAO [13,14]. There is, however, insufficient evidence for the use of X-ray pelvimetry in determining the best delivery type in women [15].

Even though concerns regarding pregnancy and delivery after undergoing PAO are common, only few studies have been performed in this regard. Both Flückiger et al. and Bartosiak et al. showed an increased risk of cesarean sections after PAO. Furthermore, they found that in 30–50% of cases, obstetricians performed a cesarean section due to expected complications during vaginal delivery after PAO [14,16]. Valenzuela et al., on the other hand, showed similar rates of cesarean sections in women after PAO compared with the US cesarean section birth rate of 25% [17].

Overall, the literature regarding pregnancy and child delivery after PAO is scarce. Thus, our study’s aim was to investigate patient and physician decision making regarding pregnancy as well as peripartum complications in women who had previously undergone PAO.

## 2. Materials and Methods

### 2.1. Patients

This study was approved by the institution’s ethics committee (EA1/052/21). All female patients undergoing PAO for a primary diagnosis of symptomatic DDH between January 2015 and June 2017 at a single institution were included retrospectively. Patients who underwent PAO for indications other than symptomatic DDH, prior surgery on the ipsilateral hip joint or inadequate pre- or postoperative radiological imaging were excluded. Demographic and perioperative data were collected using electronic medical reports and included age, gender, body mass index (BMI) and duration of surgery measured from skin incision until the completion of skin closure.

### 2.2. Preoperative Radiological Imaging

Preoperative radiological imaging included standing anterior–posterior pelvis and 30° abduction functional radiographs. In all included hips, at least one radiological abnormality, including a lateral center-edge angle of Wiberg (LCEA) less than 25°, acetabular inclination (AI) greater than 10°, an anterior center-edge angle (ACE) as described by Lequesne and de Seze of less than 25° and a femoral head extrusion index (FHEI) as described by Heyman and Herndeon of greater than 26%, was found [18,19,20]. Preoperative femoral head congruency as determined by 30° abduction functional radiographs was good in all hips.

### 2.3. Surgical Technique

All PAOs were performed as previously described by one of two experienced orthopedic surgeons specialized in hip surgery [4]. An anterior approach was used, and acetabular reorientation was achieved under fluoroscopic guidance. The acetabular fragment was fixated either by three to four screws (4.5 mm) or four to five unthreaded K-wires (2.5 mm) introduced through the iliac crest. Iliac osteotomy was performed as previously described, and the supra- and retroacetabular gap was filled with allogenic bone grafts [21]. Intraoperatively, a normalization of the LCEA to 30°, AI below 10° and an FHEI of between 10 and 25% were targeted.

### 2.4. Postoperative Radiological Assessment

**Figure 1:**
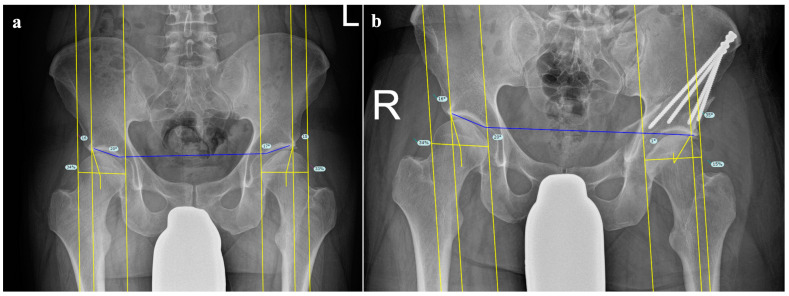
Radiological measurements of a dysplastic hip treated with PAO (**a**) preoperatively and (**b**) postoperatively. The LCEA, TA and FHEI were significantly improved pre- to postoperatively.

Postoperative assessment was performed using standing anterior–posterior pelvis radiographs before discharge and at the three-month follow-up. Parameters relevant for DDH including the LCEA, Tönnis angle (TA) and FHEI were measured by two orthopedic residents both trained by a senior orthopedic surgeon (Figure 1).

### 2.5. Survey

Pregnancy-related parameters were evaluated by a questionnaire. All patients were contacted via mail and asked to complete a survey which included both PAO- and pregnancy-related questions. The primary outcome was the birth delivery method. Secondary outcomes included birth weight, duration of pregnancy before delivery and pregnancy-related complications. Deliveries before 37 weeks were defined as preterm, while deliveries before 34 weeks were defined as early preterm [22]. PAO-related questions included patient satisfaction with the procedure and whether the patient would have the procedure performed again. Furthermore, the short version of the International Hip Outcome Tool (iHOT-12) was used to measure health-related quality of life and changes after PAO [23].

### 2.6. Statistical Analysis

Data analysis was performed using SPSS version 27 (SPSS Inc., Chicago, IL, USA). Means and standard deviations were calculated for continuous parameters. For nominal parameters, frequencies were analyzed. For the comparison of parametric values, Student’s t-test was used, and for the comparison of non-parametric values, the Mann–Whitney U test was used. The statistical significance level for all tests performed was *p* < 0.05.

## 3. Results

**Figure 2:**
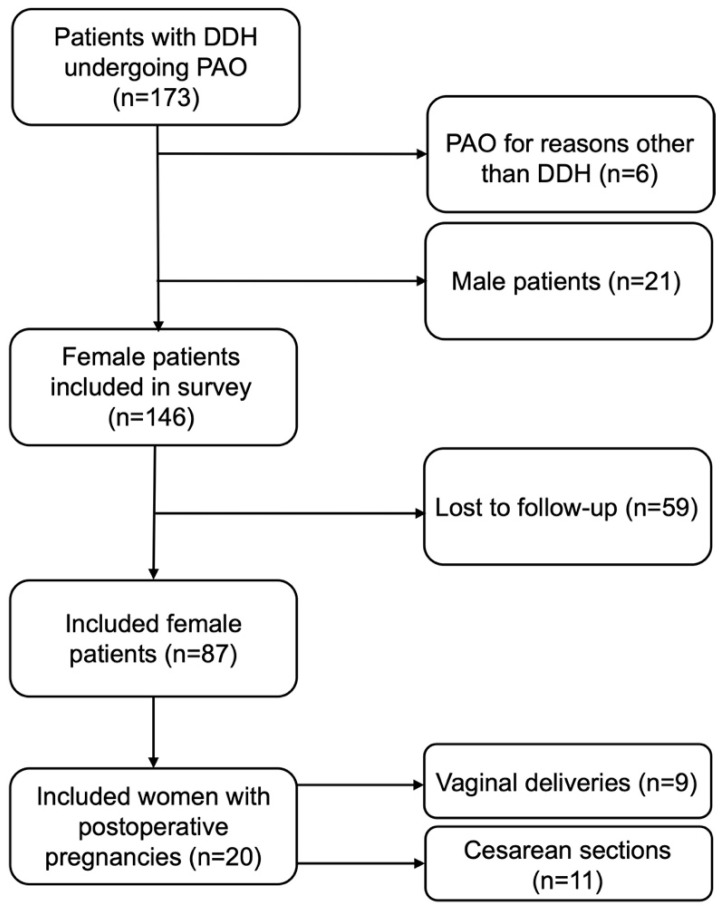
Flow chart of patient inclusion. DDH, developmental dysplasia of the hip; PAO, periacetabular osteotomy.

A total of 173 patients with 202 PAOs were identified. Six PAOs were excluded due to diagnoses other than DDH. Of the remaining patients, 146 patients with 165 PAOs were female. In total, 87 patients (59.6%) completed the PAO-related questions, while 57 patients (39.0%) completed the pregnancy-related questions, of whom 20 (22.7%) gave birth to 26 children after PAO (Figure 2).

One woman gave birth to twins, and one woman had one child before PAO. The mean patient age at the time of surgery was 28.3 ± 7.8 years. The mean BMI was 24.6 ± 4.4 kg/m^2^. Pre- and postoperative radiological parameters are shown in Table 1.

The mean overall follow-up time was 5.3 ± 0.8 years. Of all women who completed the PAO-related survey, 70 of 87 (80.4%) reported being satisfied with the surgery’s outcome. Seventy-one (81.6%) women reported they would have the procedure performed again. Of the women who had a child after PAO, 17 (85.0%) reported being satisfied with the surgery’s outcome, and 16 (80.0%) would have the procedure performed again. The mean pre- and postoperative iHOT-12 scores were 45.7 ± 23.1 and 76.5 ± 22.0, respectively, with a mean difference between pre- and postoperative scores of −30.8 ± 27.3. While the overall preoperative iHOT-12 scores did not differ significantly between satisfied and unsatisfied patients (44.1 ± 23.6 vs. 54.4 ± 21.9, *p* = 0.490), there were significant differences in postoperative iHOT-12 scores (82.8 ± 16.6 vs. 40.6 ± 11.3, *p* = 0.007) and between the pre- and postoperative scores (38.7 ± 19.9 vs. 13.9 ± 20.7, *p* = 0.001). Patients who were not satisfied with the procedure reported significantly lower values for all items except pain (Table 2). Overall, 16 of 57 (28.1%) patients reported that a history of PAO affected their decision to have a child or consider becoming pregnant.

The mean follow-up time for women who had a child after PAO was 5.3 ± 0.8 years. Pregnancy-related parameters for the first pregnancy after PAO are shown in Table 3.

The mean time before the first child’s birth was 2.9 ± 1.3 years. The mean pregnancy duration before delivery was 38.8 ± 3.3 weeks. Four children were born preterm, two of whom were early preterm. Eight patients had a second child within 3.4 ± 1.4 years after PAO. One of these children was born preterm. Five (25.0%) patients reported pregnancy-related complications including elevated blood pressure (15.0%), diabetes (10.0%) and pre-eclampsia (10.0%).

In six (30.0%) patients, labor had to be induced. Ten (50.0%) patients had an epidural anesthesia. Eleven (55.0%) patients underwent cesarean section, of which eight were due to child-related complications (breech presentation, fetal status, failure to progress with labor). Three (27.3%) patients stated that a cesarean section was performed due to their history of PAO. When only looking at first-time singleton pregnancies, the rate of cesarean section was 55.6% (10/18).

Six (23.1%) newborns required intensive care unit stays after delivery, five of whom had been delivered via cesarean section. The mean birth weight was 3107.7 ± 851.1 g for the first child and 3392.5 ± 778.8 g for the second child after PAO. In first children delivered vaginally, the mean birth weight was 2996.8 ± 877.8 g, while in first children delivered via cesarean section, the mean birth weight was 3072.5 ± 9 03.26 g.

Four (20.0%) patients reported that their obstetrician expressed concern due to their history of PAO. Six (30.0%) patients reported that their obstetrician recommended a certain type of delivery due to their history of PAO, with three obstetricians recommending cesarean section and three recommending vaginal delivery.

## 4. Discussion

Even though PAO is an established procedure for joint-preserving surgical therapy of DDH in young women, its influence on postoperative pregnancies has hardly been studied. We therefore performed a questionnaire-based analysis of the course of pregnancies in women with a history of PAO and found an increased rate of cesarean sections of 55.0% in the first pregnancy after PAO, with the reported reason for this delivery type being a history of pelvic osteotomy in three cases.

Prior to PAO, female patients are often concerned about the intervention’s influence on later pregnancies and child delivery. To date, only very few reports with low patient numbers concerning this issue exist, and thus evidence-based recommendations are lacking. In line with previous reports, our analysis indicated that patients with a history of PAO have a higher risk of delivering a child by cesarean section compared with the general population [13,15]. In our cohort, 55.6% of woman delivered their first child after PAO by cesarean section in contrast to an overall rate of 29.7% in German women [24]. This rate did not change significantly after excluding patients with twin pregnancies or prior pregnancies. In our study, the mean birth weight of first children after PAO was 3107.7 g, which is in line with the previous report of Bartosiak et al. [16] but lower compared with the mean birth weight in Germany [25].

While cesarean sections need to be performed due to medical indications such as labor dystocia, fetal malpresentation or abnormal fetal heart rate tracing, they may also be planned as an elective procedure for reasons such as the obstetrician’s or the patient’s preference [26,27]. In our analysis, four women reported that their obstetrician was concerned, and three obstetricians recommended a cesarean section due to a history of PAO. This is in line with a previous report, in which a history of pelvic osteotomy was named as a reason for cesarean section in 6 of 20 births [16]. Reasons for obstetricians’ uncertainty regarding the optimal birth procedure may be the lack of evidence of morphological changes in the birth canal after pelvic osteotomy, and of pelvic parameters predicting failure of vaginal delivery. Generally, a mid-pelvis diameter of less than 95 mm has previously been regarded as the low threshold value for a vaginal delivery [28]. To date, only three studies investigated birth canal changes after PAO. While Flückiger et al. studied pelvic radiographs and found no significant changes in 17 women, Trousdale et al. measured pelvic diameters in MR images of 7 women before and after PAO and also found no significant changes [13,14]. Loder et al. found a transverse mid-pelvis diameter below the threshold for cesarean section in 20% of the included 30 patients’ radiographs [29]. However, they investigated several different types of pelvic osteotomy and did not find a significant decrease in the mid-pelvis diameter in either of the two included PAO procedures. Furthermore, it has been shown that there is not enough evidence for the use of X-ray pelvimetry in deciding on the mode of delivery. While women receiving pelvimetry have a higher risk of cesarean section being performed, there is no reduction in fetal mortality or morbidity rates [15].

Cesarean sections are overall safe procedures and are associated with a low incidence of perioperative complications. Thus, they are routinely performed in case of maternal or fetal complications. At the same time, it has been shown that cesarean sections are associated with significantly greater mortality and morbidity rates than vaginal deliveries, which is mainly due to the high risk of hemorrhage and of infection [30,31,32]. Besides these short-term risks, there are concerns regarding long-term risks such as placental abnormalities in future pregnancies as well as the necessity to deliver subsequent children via cesarean section, which in turn leads to an increase in surgical risk [33,34]. For the fetus, cesarean section is safer, but there are risks mostly in terms of respiratory complications as well [35].

Since the 1990s, cesarean rates have been increasing throughout the world. However, due to a lack of evidence for a concomitant decrease in maternal or fetal morbidity or mortality, it is assumed that cesarean delivery is overused [36]. Especially due to the long-term risks associated with cesarean section and repeat cesarean delivery, much effort has been devoted to safely reducing the primary cesarean delivery rate. While to achieve this, a number of approaches are needed, it is necessary to provide evidence-based guidelines with specific recommendations. Prior pelvic reconstructive surgery is often mentioned as an indication for cesarean section, yet to date, there is no literature supporting this claim [37].

Some limitations need to be discussed. First, the percentage of patients who replied to the survey was 39.0%, and only 20 of these patients gave birth to a child after PAO. This may have caused a nonresponse bias. Additionally, all analyzed data were self-reported by the patients as we did not have access to any pregnancy-related patient charts. Furthermore, the low patient number did not allow for any advanced statistical analysis or for any final conclusions to be drawn. However, our results still add to the small existing body of literature and may—especially with the threshold for performing procedures such as PAO steadily lowering—lead to larger database studies being conducted [38].

Overall, our results add to the small existing body of literature, providing evidence of an increased rate of cesarean sections in patients with a history of PAO. As, to date, there is no reliable evidence for the necessity of delivering via cesarean section after pelvic osteotomy, there is a need for high-quality studies investigating the benefit of cesarean sections in this patient group. Especially since cesarean sections are associated with higher morbidity and mortality compared with vaginal deliveries, evidence-based recommendations are urgently needed.

## Figures and Tables

**Table 1:** Pre- and postoperative radiographic parameters in women who had a child after PAO.

Parameter	Preoperative	Postoperative	*p*-Value
LCEA (°)	16.4 ± 6.4	29.7 ± 6.4	0.031 *
TA (°)	13.2 ± 6.8	1.7 ± 7.4	0.011 *
AWI	0.4 ± 1.5	0.5 ± 0.2	0.013 *
PWI	0.8 ± 0.2	0.8 ± 0.2	0.396
FHEI	0.2 ± 0.1	0.1 ± 0.1	<0.001 *

Results are presented as means and standard deviations. Statistically significant *p*-values are marked with *. LCEA, lateral center-edge angle of Wiberg; TA, Tönnis angle; AWI, anterior wall index; PWI, posterior wall index; FHEI, femoral head extrusion index.

**Table 2:** Postoperative iHOT-12 scores for each item in patients who reported being satisfied with the procedure and patients who were not satisfied.

iHOT-12	Patients Satisfied with PAO (*n* = 17)	Patients Not Satisfied with PAO (*n* = 3)	*p*-Value
Pain	7.6 ± 3.4	4.0 ± 1.0	0.054
Getting up and down	8.9 ± 1.7	3.0 ± 1.0	0.002 *
Walking long distances	8.2 ± 2.4	4.7 ± 1.2	0.028 *
Grinding, catching, clicking	8.7 ± 3.5	4.3 ± 2.3	0.019 *
Pushing, pulling, lifting, carrying	8.1 ± 2.1	3.7 ± 0.6	0.004 *
Concern about changing directions during sports	7.8 ± 2.6	4.0 ± 2.6	0.040 *
Pain after activity	8.1 ± 2.1	4.3 ± 1.5	0.019 *
Picking up or carrying children	8.1 ± 2.8	4.0 ± 1.7	0.047 *
Sexual activity	8.9 ± 1.8	4.3 ± 2.3	0.012 *
Awareness of disability in hip	8.0 ± 4.0	4.0 ± 2.6	0.012 *
Maintaining fitness level	8.4 ± 1.8	3.3 ± 3.5	0.012 *
Distraction	8.5 ± 2.1	5.0 ± 2.6	0.040 *

Statistically significantly different p-values are marked with *. iHOT, International Hip Outcome Tool; PAO, periacetabular osteotomy.

**Table 3:** Pregnancy-related parameters for the first pregnancy after PAO. Mean values are presented with the standard deviation.

Type of Delivery	Preoperative
Cesarean section Vaginal	11/20 (55.0%) 9/20 (45.0%)
Gestation (weeks)	38.8 ± 3.3
Preterm births	4/20 (20.0%)
Birth weight (grams)	3107.7 ± 851.1

## Data Availability

The data presented in this study are available on request from the corresponding author. The data are not publicly available due to patient privacy.
